# Burden of Respiratory Syncytial Virus Associated Severe Pneumonia in Hospitalized Children

**DOI:** 10.1155/2021/8269400

**Published:** 2021-06-30

**Authors:** Madhusha Gonapaladeniya, Thushari Dissanayake, Guwani Liyanage

**Affiliations:** ^1^Department of Microbiology, Faculty of Medical Sciences, University of Sri Jayewardenepura, Sri Lanka; ^2^Department of Pediatrics, Faculty of Medical Sciences, University of Sri Jayewardenepura, Sri Lanka

## Abstract

Respiratory syncytial virus (RSV) is a leading cause of severe respiratory infections. We examined the burden of RSV-associated severe community-acquired pneumonia among hospitalized children and factors that predict RSV etiology. A hospital-based prospective study examined children below five years of age admitted with radiologically confirmed severe or very severe pneumonia in two tertiary care centers in Sri Lanka. Nasopharyngeal secretions (NPS) were tested for 19 viruses by multiplex RT-PCR. Univariate and multivariate analysis was performed to determine whether RSV etiology could be predicted based on clinical, sociodemographic, environmental, radiological, and laboratory parameters. A total of 108 children with severe or very severe were included in the study. At least one virus was found in NPS in 92.5% of children. Forty-six children had RSV (+) pneumonia. Mean RSV proportion was 42.6% (95% CI: 33.1-52.5%, *p* value = 0.149). RSV as a single virus was found in 41.3% (19/46). The children with RSV (+) pneumonia were younger (*p* = 0.026) and had lower C-reactive protein (*p* = 0.003) and household crowding (*p* = 0.012) than the RSV (-) group, after controlling for confounding covariates. In conclusion, the present study demonstrated that respiratory syncytial virus was the commonest virus associated with CAP in children under five years. Younger age, crowded housing, and lower C-reactive protein levels were predictors of severe RSV-associated pneumonia.

## 1. Introduction

Respiratory syncytial virus (RSV) is a leading cause of severe respiratory infections, predominantly in young children [1]. No effective and safe medication or vaccine for RSV infections is available at present. The only preventive method considered is prophylaxis with palivizumab, an RSV-neutralizing monoclonal antibody administered in high-risk groups during epidemics [2].

The recent multisite PERCH study on children below five years with community-acquired pneumonia reported that RSV infection accounts for the greatest etiological fraction among a broad array of other pathogens [3]. Despite the epidemiological importance, there is limited evidence on the impact of RSV-associated pneumonia in resource poor settings. Further, factors that predict etiological agents of community-acquired pneumonia are being explored in many settings as such information is beneficial to deliver appropriate therapy, reduce antibiotic consumption, and avoid costly microbiological tests [4, 5]. We examined the burden of RSV associated with severe community-acquired pneumonia (CAP) among hospitalized children in Sri Lanka and factors that predict RSV etiology.

## 2. Methods

### 2.1. Study Setting

This descriptive analysis is based on a study carried out among children aged three to 60 months with severe or very severe community-acquired pneumonia admitted to two tertiary care centers located in the Colombo district, in Sri Lanka, from June 2018 to April 2019. The institutional ethics review committee approved the protocol of this study (USJP/FMS/ERC/60/17).

### 2.2. Data Collection Procedure

Each consecutive patient with physician-diagnosed pneumonia was screened. Infants less than three months were not recruited to avoid inclusion of late-onset group-B streptococcal (GBS) disease, defined as GBS infection from day 7 to day 89 of life [6]. Patients who met the inclusion criteria for severe and severe CAP and agreed to participate by signing an informed consent form were included in the study. Patients with any of the following were excluded: immune deficiency, congenital heart disease, congenital airway, lung malformations or dysplasia, foreign bodies in the airway, illness of >7 days before admission, and delay sample collection for more than 48 hours after admission.

Demographic characteristics, medical history, vital signs, and clinical details were recorded on a pretested data collection form. Nasopharyngeal aspirates and blood samples (complete blood count, C-reactive protein, and blood culture) were taken on admission or within the first 48 hours. Nasopharyngeal aspirates were collected with a recommended mucus extractor by the investigators and stored at -80°C until analysis. As per the manufacturer's instructions, the NPS same sample was used for both viral and bacterial detections by multiplex-PCR. Samples were transported to the reference laboratory in ice within two hours. A commercially available transport medium to stabilize RNA and DNA of both viruses and bacteria was used. The samples were stored at -80C until processed. Chest X-rays were reported by a radiologist who was blinded to the clinical details. The X-ray reports were categorized as having consolidation ± pleural effusion, interstitial infiltrates without pleural effusion, and no consolidation/infiltrate/effusion [7]. Children with negative, uninterpretable, or suboptimal chest x-rays were not included in the final sample. All children were followed up till discharge.

### 2.3. Community-Acquired Pneumonia Case Definition

World Health Organization (WHO) criteria that define pneumonia were modified to exclude children with asthma exacerbations and bronchiolitis: the presence of fever (before or after hospital admission), cough (±sputum), abnormal chest signs (localized crackles or increased vocal resonance or reduced breath sounds), and tachypnoea [3, 8]. The presence of moderate respiratory distress (lower chest wall indrawing) in children with pneumonia was categorized as severe pneumonia. The presence of any one or more of the following features in children with severe pneumonia was considered very severe pneumonia: central cyanosis, severe respiratory distress (grunting or labored breathing), inability to drink or breastfeed, or persistent vomiting, altered consciousness, convulsions, and respiratory failure. Previous WHO-defined severity classification (2005) was used instead of recently modified WHO version (2013) to define children with severe and very severe pneumonia [8, 9]. WHO-defined 2005 classification was considered more appropriate as it enabled us to compare our findings with those of large multicenter studies from low, middle, and emerging economies that used the same classification [3, 10].

### 2.4. Laboratory Analysis

C-reactive protein, complete blood count, and blood culture were analyzed at the laboratory at the two study sites. Reference ranges for total white blood cell count and C-reactive protein were 4-11 × 10^9^/L and <6 mg/dL. Blood cultures were processed using an automated analyzer (BD BACTEC™). Both laboratories at the study sites are under one parent organization and follow common standards and guidelines. Necessary standards of performance are clearly defined, and adherence to the protocol is monitored. Thus, we assumed that the results were accurate and comparable between the two laboratories. Multiplex real-time- polymerase chain reaction (RT-PCR) assay was performed for 19 viruses and five bacteria (fast-track diagnostics respiratory pathogens 21 plus; Fast-track Diagnostic, Luxembourg) at the Research Laboratory of Faculty of Medical Sciences, University of Sri Jayewardenepura. Bacterial detection in NPS was considered colonization, and quantification was not done.

### 2.5. Data Analysis

Continuous variables were reported as the mean, standard deviation, median, and range. Categorical variables were expressed as frequencies and percentages. Univariate logistic regression was performed to examine the potential predictor variables: age (2 years vs. >2-5 years), gender, birth weight (low birth weight < 2.5 kg), maturity (preterm < 37 weeks), exposure to cigarette smoke, household crowding (defined as ≥two other occupants in child's bedroom), duration of exclusive breastfeeding, maternal education (primary vs. secondary and tertiary), income (>40,000LKR vs. ≤40,000), previous history of wheezing, clinical details (body temperature, respiratory rate, pulse rate, and oxygen saturation in the air on admission (≥92% vs. <92%)), gastrointestinal symptoms (vomiting ± loose stools, etc.), biological parameters (CRP, total white blood cell count, and neutrophil count), CXR findings (end-point consolidation or interstitial infiltrates), duration of hospitalization, and high dependency care. CRP level was dichotomized as <20 mg/L and ≥20 mg/L according to a previous literature [4]. We used all the variables with low *p* values (<0.1) in the multivariate logistic regression. SPSS software version 22.0 was used for data analysis.

## 3. Results

### 3.1. Baseline Characteristics of the Study Population

All children aged three months to 5 years with physician-diagnosed pneumonia were screened during the study period (*n* = 201). The majority were direct admissions (180/201), while others were referrals. Subsequently, 131 eligible children with severe or very severe CAP were invited to participate. Reasons for noneligibility were nonsevere pneumonia (*n* = 56), onset of illness > 7 days (*n* = 5), and chronic disease (*n* = 9). Fifteen were excluded due to refused consent for participation and eight due to delayed sample collection. The response rate was 82.4%.

None of the children had received the pneumococcal vaccine as pneumococcal vaccine is not introduced yet, in the public-funded immunization program in Sri Lanka. Most children were pretreated with antibiotics before admission (82.4%). Overall, very severe pneumonia was found more among the younger age group (less than 24 months) than the older age group (between 24 and 60 months) (61.3% vs. 53.3%); however, the difference was not significant (*p* = 0.806). All children received either intravenous or oral antibiotics. There was no mortality in the study group. At least one virus was found in NPS in 92.5% of children. Forty-six children had RSV (+) pneumonia. Mean RSV proportion was 42.6% (95% CI: 33.1-52.5%, *p* value = 0.149). Of that, RSV as a single virus was found in 41.3% (19/46). RSV proportions were significantly different between age strata (71.4% in ≤2 years). Pseudomonas aeruginosa was isolated in one blood culture; none of the other cultures were positive.

### 3.2. Comparison between RSV (+) and RSV (-) Pneumonia

Comparison of RSV (+) and RSV (-) pneumonia related to sociodemographic, environmental, clinical, biological, and radiological parameters are presented in Table 1. Children with RSV (+) pneumonia were younger (<2 years) and had lower CRP (<20 mg/dL) and interstitial infiltrates in the CXR than RSV (-) pneumonia. None of the RSV (+) pneumonia had effusions. The need for intravenous antibiotic treatment was significantly higher in the RSV (-) group compared to the RSV (+) group (*p* = 0.027). Duration of hospital stay and need for high dependency care were equal in both groups. Comparison of children with RSV (+) and RSV (-) pneumonia is shown in Table 1.

**Figure 1:**
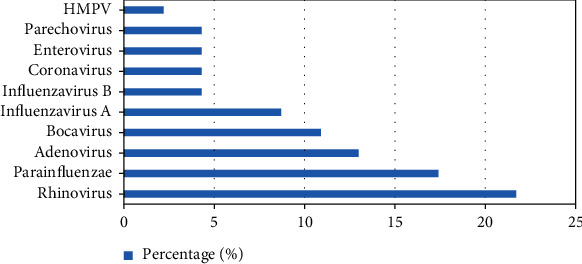
Distribution of other viral pathogens in NPS of children with RSV (+) pneumonia.

**Figure 2:**
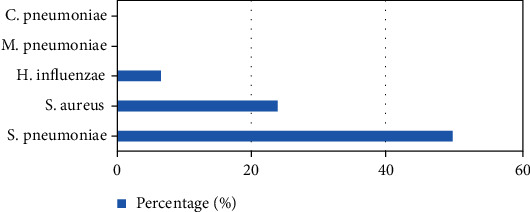
Distribution of bacteria in NPS of children with RSV (+) pneumonia.

Figures 1 and 2 depict the codetected viruses and bacteria in NPS. Rhinovirus (RV) was the commonest virus concomitantly detected in RSV (+) CAP (21.7%). However, RV fraction was significantly higher in RSV (-) CAP (41.9%) (*p* = 0.039). Bacteria were found in 66.7% of total NPS. Detection of pneumococci was not different in RSV (+) and RSV (-) CAP (*p* = 0.439). Also, pneumococcal detection was similar between rhinovirus (+) and rhinovirus (-) children (*p* = 0.079).

### 3.3. Predictors of RSV (+) Pneumonia with Multivariate Regression Analysis

Multivariate regression was performed to examine the predictors of RSV-associated pneumonia. All factors showing *p* < 0.1 in the univariate analysis were considered for inclusion. After excluding collinearity, we obtained the best fitting model, and predictors of RSV (+) pneumonia are summarized in Table 2. According to the model, age, crowding, and CRP were added significantly to the model/prediction, but not the CXR findings. The odds of having RSV (+) pneumonia were 4.711 times greater for children with CRP of <20 mg/dL as opposed to ≥20 mg/dL.

## 4. Discussion

This prospective observational study examined the burden or the predictors of RSV associated CAP among hospitalized children. Most notably, viruses were the leading organisms detected. RSV was the commonest virus that agrees with previous studies from temperate and tropical countries [3, 11]. Influenza virus had a lesser role as the likely cause of pneumonia in children in this study, comparable to other studies in the Asian subcontinent [3, 10]. Bacterial pathogens were isolated only in one blood sample. The lower rate of bacterial isolation could be due to pretreatment with antibiotics and inherently low blood culture yield in CAP. Therefore, perhaps, the comparison of actual bacterial coinfection rate in RSV (+) and RSV (-) pneumonia could not be accurately determined with the study design. In a study among children less than five years in the UK, the invasive pneumococcal disease was seen more with influenza than with RSV pneumonia [12]. Similarly, in a multicenter study, infants with RSV infections were at significantly lower risk of serious bacterial infections than infants with non-RSV infections [13].

Importantly, we observed that age, CRP, and crowding were significant predictors of RSV (+) CAP when controlled for confounding covariates. Frequently, CRP is considered helpful for the physician to differentiate viral from bacterial infection. It is a low-cost test that is widely available. Our findings showed that it is perhaps a reasonably good tool that could be utilized along with other factors to differentiate RSV (+) and RSV (-) pneumonia. In addition to the significant mean difference of CRP between RSV (+) and RSV (-) pneumonia, a cut-off level of 20 mg/L contributed to the regression model more than the other predictor variables. Similar results had been reported previously [3, 4]. A multisite, international case-control study (Pneumonia Etiology Research for Child Health (PERCH)) in nine sites in seven countries reported a sensitivity of 77% and specificity of 82%, for a CRP cut-off of <37.1 mg/L, in differentiating RSV-associated pneumonia [3].

A significant association between RSV (+) pneumonia and household crowding (defined as the number of occupants in the child's bedroom) was evident. Crowding is a risk factor for a range of infectious diseases and, most importantly, for most respiratory tract infections [14]. It can facilitate the spread through viral shedding. Typically, the highest shedding occurs early in infection, and high viral loads can be passed to close contacts [15]. Crowding has been previously reported as a significant risk factor for the high prevalence and severity of RSV disease in infants and young children [16, 17]. When evaluating crowding, ages and the number of others in the house and sharing the same bedroom are important considerations. Thus, perhaps, the comparison between studies is difficult because of the variability of definitions used for crowding.

In the present study, CXR findings were not a significant predictor when controlled for confounding covariates. Yet, the number of children with CXRs showing alveolar infiltrates was high among the RSV (-) group. Thus, it can be speculated that higher fraction of alveolar infiltrates among RSV (-) children indicated greater fraction of bacterial coinfection. A previous research by Diniz et al. found alveolar infiltrates in all patients who had confirmed bacterial, fungal, and mixed infections in a sample of preterm infants, while the interstitial infiltrates were found with viral lower respiratory infections [18]. However, in general, radiographic findings are not useful in differentiating bacterial from a nonbacterial cause, reliably [19].

In general, disease severity and hospitalization rate impact the healthcare burden. Also, it is shown that children underlying comorbidities have more severe disease, needing intensive care [20]. In the present study, the disease severity was not significantly different between the RSV (-) and RSV (+) groups. The hospital stay and high dependency care were almost equal between the two groups in the present study. One explanation for this finding could be that not including children with comorbidities. Besides, we analyzed only the hospitalized children with severe CAP; therefore, community-based studies on RSV epidemiology and outcomes would perhaps be ideal for estimating the true impact of RSV-associated pneumonia in our setting.

This study should be interpreted with few limitations. Children with pneumonia directly admitted to the intensive care units were not enrolled. In the absence of a validated definition for pneumonia, some cases may have been erroneously diagnosed as pneumonia, or true cases could have been excluded. Also, case definition included positive results in chest X-rays to assume that all children recruited had a lung infection and to avoid inclusion of children with viral wheezing and bronchiolitis. However, we may have overlooked RSV-positive pneumonia with negative CXR as radiological changes lag behind clinical symptoms. However, to minimize errors in diagnosis, we incorporated additional criteria to the WHO clinical definition of pneumonia. Also, we added strength to the diagnosis by recruiting only the children with radiologically confirmed pneumonia.

## 5. Conclusion

We demonstrated that the respiratory syncytial virus was the commonest virus associated with CAP in children under five years. Severe RSV infection is characteristically associated with younger age, crowded housing, and lower C-reactive protein levels among hospitalized children.

## Figures and Tables

**Table 1:** Comparison of children with RSV (+) and RSV (-) pneumonia.

	RSV (+) (*n* = 46)	RSV (-) (*n* = 62)	*p* value	OR
*Sociodemography & environmental risk factors*				
Age < 2 years	81.4	48.4	0.004	3.394 (1.464-7.869)
Gender, male	39.1	46.8	0.429	1.367 (0.630-2.965)
Birth weight < 2.5 kg	10.8	20.9	0.171	0.460 (0.151-1.367)
Maturity < 37 weeks	8.6	9.6	0.862	1.125 (0.298-4.241)
Cigarette smoke exposure	26.1	27.4	0.588	1.283 (0.520-3.169)
Household crowding	80.4	56.5	0.064	2.400 (0.951-6.058)
Exclusive breast feeding < 4 m	28.5	17.5	0.217	1.880 (0.690-5.120)
Maternal education ≥ secondary	60.86	74.2	0.621	1.339 (0.412-4.258)
Monthly income > 40, 000LKR	39.1	40.3	0.480	1.355 (0.583-3.153)
Daycare attendance	5.7	5.3	0.926	1.091 (0.173-6.875)
*Clinical features and severity*				
Wheezing in the past	13.0	8.1	0.402	1.710 (0.488-5.991)
Temperature (°F), mean (SD)	102.08 (1.24)	102.17 (1.34)	0.700	0.943 (0.700-1.270)
Oxygen saturation < 92%	39.2	35.5	0.698	0.856 (0.389-1.882)
Gastrointestinal symptoms	21.7	12.9	0.227	1.875 (0.676-5.204)
Very severe pneumonia	58.69	51.6	0.465	0.751 (0.348-1.621)
High dependency care	2.23	3.23	0.744	1.500 (0.132-17.06)
Hospital stay (days), median (Q1, Q3)	5 (3.0, 8.25)	6 (3.75, 8.25)	0.778	0.989 (0.917-1.067)
*Biological and radiological parameters*				
CRP < 20 mg/L	63.04	32.78	0.002	3.497 (1.567-7.802)
Total WBC (10^9^/L), mean (SD)	13.42 (6.19)	15.38 (7.55)	0.154	0.960 (0.906-1.016)
Neutrophils (10^9^/L), mean (SD)	7.81 (5.83)	9.60 (6.40)	0.141	0.952 (0.892-1.017)
CXR interstitial infiltrates	73.9	50.0	**0.013**	2.833 (1.241-6.467)

Descriptive statistics are expressed as percentages unless otherwise indicated. CXR: chest X-ray; CRP: C-reactive protein; LKR: Sri Lankan rupees; WBC: white cell count.

**Table 2:** Predictors of RSV (+) pneumonia.

	Regression coefficient *β*	Exp (*β*)	95% CI for Exp (*β*)		*p* value
			Lower	Upper	
Age (<2 years)	1.207	4.243	1.154	9.689	0.026
Household crowding	1.445	4.243	1.374	13.11	0.012
CXR (interstitial infiltrates)	0.731	2.078	0.740	5.837	0.165
CRP < 20 mg/L	1.550	4.711	1.678	13.22	0.003

Log-likelihood ratio = 99.04; Nagelkerke *R*^2^ = 0.338; *X*^2^ = 26.98; *p* < 0.001.

## Data Availability

Data is available at a reasonable request from the corresponding author.
